# Supplementary material for: It’s made a really hard situation even more difficult: The impact of COVID-19 on families of children with chronic illness
Source: PLoS One. 2022 Sep 1;17(9):e0273622. doi: 10.1371/journal.pone.0273622 (PMC9436103; doi:10.1371/journal.pone.0273622)
Supplement: S1 Table — (DOCX) [file pone.0273622.s001.docx]

Supplementary Table 1: The perceived impact of COVID-19 - illustrative quotations of longer length.

| COVID-19 specific concerns | |
| --- | --- |
| Summary | Quote |
| The severe impact of viruses on a chronically ill children | “Even as news came in that kids don’t seem to get sick with it so much, there was enough data online that said most kids don’t, but some kids do... So that was a really nerve wrecking experience.” (ID 06, child with severe asthma) |
| Prolonged isolation | We're more aware of where we’re going and how we’re acting in the community. We are very conscious and we’ve been masking for a long time. We've still got the hand sanitiser everywhere and we are still being cautious.” (ID 10, child with epilepsy) |
| The impact of parents’ developing COVID-19 on the child’s care needs | “I am very, very worried about if [my husband] and I got it - what would happen to [my daughter]. We do all of [her] care - her 11 hours of dialysis every night, we have to count every milliliter she's drinking, we have to double boil her puree, we have to weigh all her food, it’s extreme okay and I am extremely scared about what would happen, if [my husband] and I went down - what would happen to [my daughter]?” (ID 09, child with end stage kidney failure) |
|  | You know as a parent …they need you. Like it doesn’t work if we don’t have parents to keep this going forward. It’s that risk, even if she’d be fine if she got it, if one of us got it, it would be disastrous.” (ID 10, child with epilepsy) |
| Chronically ill children advocated for the vaccine | “I think she was the most excited I’ve ever seen a child about anything when we got our vaccination appointment. She was really excited because in her mind that was a big thing to sort of mark …that it will be over.” (ID 10, child with epilepsy) |
| COVID-19 interruptions to medical care | |
| Summary | Quote |
| Cancellation of clinics | “His last two operations they thought he had an anaphylactic reaction. So [he had an] allergy clinic [in] March, so they cancelled it then they made a new one, they cancelled it again… Then he had to have surgery in the beginning of August …and they wanted to do all the [allergy] testing before that, but they decided not to because of COVID. But then he had the reaction again! So then they decided, “Oh actually, you do need to come to [the allergy] clinic!” (ID 08, child with mid-aortic syndrome) |
| Delays for critical scans and surgeries | “They said that there was a big delay on being able to provide that [SPECT scan] because the radioactive substance that’s injected in the kids, there was a shortage of that in Australia because of COVID.” (ID 13, child with epilepsy (cortical dysplasia)) |
|  | “[the surgeon said] ‘What do you want to do? We could probably squeeze her in before elective surgeries and all those things are closed and cancelled [end of March]. But obviously we've got the worry of COVID. What do you want to do?’ And I'm like I don’t know? It’s a very hard decision to make. I really agonised over it and in the end … it got postponed. …The option [didn’t become available again until] July. If this [surgery] had worked, [my daughter] would be getting a transplant.” (ID 09, child with end stage kidney failure) |
| Interstate border closures restricted surgeries at centers of excellence | “When we spoke to them about the possibility of having surgery interstate, the body language of the doctor was very defensive. Whereas I think if it wasn’t for COVID we would be able to access that service much more easily.” (ID 13, child with epilepsy (cortical dysplasia)) |
| Telehealth was more convenient | “We've had neurology, respiratory, bronchiectasis, pediatrics, all on telehealth. And it’s been great. Yes. Really good. So much easier for me and for him because it really gets very draining going into the hospital for all of the appointments all of the time.” (ID01, child with cerebral palsy) |
| Telehealth supported interstate collaborations for complex cases | “So all the appointments with the Melbourne doctors have been by telehealth, which has been great. So there’s been a really nice collaboration between the Sydney doctors and the Melbourne doctors in terms of sharing of resources and that’s definitely been amazing to have access to that service via telehealth.” (ID 13, child with epilepsy (cortical dysplasia)) |
|  | “We were able to pivot her physio and do [it] by telehealth, which worked really well. Also, if we go to speech therapy we drive half an hour each direction, which means she [misses an] hour of school. If we can do the telehealth at 8 o'clock in the morning just before school, it fits in a lot better with the day.” (ID 10, child with epilepsy) |
| Support for GP telehealth use | “For me I think telehealth was another silver lining… I think that’s been really good and that should stay because for people in my situation, not having to be physically present all the time is a blessing, whether or not there's a pandemic.” (ID 04, child with acute lymphoblastic leukemia) |
| The impact of COVID-19 interruptions on mental health | |
| Summary | Quote |
| Learning from experience | “This time around I’ve been able to manage [my mental health] a bit better because I’ve been able to put different things in place. I’ve actually taken more self care.” (ID 13, child with epilepsy (cortical dysplasia)) |
| Peer support | “Some things sprung up like Redkite started doing online coffee groups and for a while I was joining that every week. That was actually quite good for me, quite therapeutic And also the Leukaemia Foundation did some similar kind of thing. So those were actually quite good for me in that lockdown, uncertain period.” (ID 04, child with acute lymphoblastic leukemia) |
| Increased family time | “Probably one of the positive things was that things slowed down quite a bit, so we weren’t rushing to afterschool activities and there was time for us to go on family walks, family bike rides and all that sort of stuff. So that was definitely a positive of [the pandemic].” (ID 07, child with posterior urethral valves) |
|  | “We are just a tight knit family, so we got on really well. …We just adapted really well. At one stage [her brother] came up to me and was like, “This is really nice, she hasn’t been to the hospital for ages and we have all this time together”. So for them, they built this beautiful little relationship they'd not necessarily had the time together to develop because he's at school and she's just been out of the house. So… it’s quite nice because we finally got to spend time as a family.” (ID 10, child with epilepsy) |
| The multiple responsibilities of mothers | “I guess the hardest thing has just been the juggling act. Just the challenge has been trying to do my job, keep the business running, run a family, and now without any kind of babysitters or cleaners or any extra help and without school and without childcare, so that’s been the trickiest part of COVID I think for me personally.” (ID 13, child with epilepsy (cortical dysplasia)) |
|  | “I think because like a lot of families the burden of childcare has largely or exclusively fallen on the mother I’d say. So I’d say that’s mostly me that is suffering as a result of that, either the extra workload or just the awareness that the general sort of structure that I prefer to have within our family and within our life there’s had to be lots of compromises in terms of just logistically being able to juggle the competing responsibilities.” (ID 13, child with epilepsy (cortical dysplasia)) |
| Distance from friends at challenging times | “[My daughter’s] initial [cancer] diagnosis was when we were really in the middle of the pandemic. When you're upset or something terrible happens you would hug your friends. …I stopped that altogether. I had friends dropping things off to me at the hospital and they’re like I wanna give you a hug and I’m like we’re not. I don’t want you. I think personally that affected me.” (ID 11, child with acute lymphoblastic leukemia) |
| Loss of professional identity | “When you get out of the house and you go to work, you're someone different, you're the person at work. But when you're kind of just stuck in this house with a pandemic, [your child’s illness] just becomes your identity and you're a cancer parent. The mental health impact of that was a lot greater than I recognized at the time.” (ID 04, child with acute lymphoblastic leukemia) |
| Psychosocial support was lacking | “I so badly need to see a counsellor. I really needed to spend some time talking to somebody particularly at the beginning. Like at the beginning they didn’t even want the social workers to meet us in person and it’s just ridiculous. Support staff needs to be accessible, if social workers and psych and things are not around it can have a terrible impact at certain times.” (ID 05, child with pre B acute lymhoblastic leukemia) |
| Long waitlists for psychosocial support | “We're finding now waitlists are a lot longer than normal for a lot of the [psychologists], just because they've got that backlog. It makes it a little trickier to access those services as a family. …it just takes a lot of running around to try and find places with availability.” (ID 10, child with epilepsy) |
| Mental health impact of the ‘one parent, no siblings’ hospital restrictions | “Hospitals are doing what they need to do, but there's a real risk attached to it. There's a real risk and I know mental health is the problem of 2020 for everybody, but there really is such a risk attached.” (ID 05, child with pre B acute lymhoblastic leukemia) |
| Separation of parents | “When my daughter was diagnosed [with cancer] they wouldn’t let my husband come into the hospital. He had to go downstairs and stand next to a Coke machine and listen in on a call with six people discussing my daughter’s diagnosis. And there was one afternoon she almost died - like the full-on warning. The professor was in the room like, "I’m concerned, you must prepare" and like my husband is sitting down in the car park. And he couldn’t support me emotionally because he just didn’t understand where I was at with it all because he just wasn’t in the room.” (ID 05, child with pre B acute lymhoblastic leukemia) |
|  | “One of the biggest things [for] people with long term illness is you've got to stay on-board with your partner. Like the strain on your home-life is a very close second to the illness itself. [I was] sitting there crying outside the hospital saying to my husband this is a really serious moment and things are not matching and they are telling us… the conversations are starting…, and he was just oblivious to it. My husband's a reasonable man, I mean we have a good marriage, good communication, and we've mainly done well through this, but I just thought holy s**t, like at that moment if we weren’t educated people, if English wasn’t our first language, if there was any disadvantage, I don’t know what could've happened that day.” (ID 05, child with pre B acute lymhoblastic leukemia) |
|  | “A lot of things get lost in translation …and it actually builds frustration between us. There's a lot of complex information that goes back and forward on a day to day basis that doesn’t always get communicated because he is not there.” (ID 11, child with acute lymphoblastic leukemia) |
| Separation of siblings | “They love each other. So she would cry for him and he was very, very confused because of his age [4 years], and he needed to see her. When she did see him, either coming home briefly or through the doors at the hospital, her mental state was immediately better for two or three days. The boost was really noticeable and it really helped our son to settle, because …he was just very confused and very upset. He was quite problematic.” (ID 05, child with pre B acute lymhoblastic leukemia) |
|  | “The kids are only 14 months apart so they are exceptionally close. [My daughter] is desperate for his companionship. She's just grown up with him that's all she's known. So for him not being able to come into the hospital, she has been incredibly distraught. I’d say that probably had the biggest impact on the family. Because I spend so much time in hospital, this past six months have been really traumatic for [my daughter], but the gravitating impact of that has been on his emotional well being. We’ll have this connect-disconnect-connect-disconnect for him. So we've [had] unexpected behaviors from him we’ve put down to us not being together as a family and him being shuffled around quite a bit.” (ID 11, child with acute lymphoblastic leukemia) |
|  | They're the best of friends. And that was the other thing I thought - how can we say this little girl can’t see her baby sister while she's going through such a hell time in the hospital? And again, I understand, at the same time I know COVID is scary and they have to make sure everyone's safe, but you also got to think about people and family. And given [my daughter’s] trauma like, [her sibling] just calmed her right down. (ID 09, child with end stage kidney failure) |
| Challenges caring for young siblings hospital | “My husband and I were allowed in ICU …the baby [was] not allowed in ICU. We had to basically pay for my parents to stay in accommodation with us so they could help look after the baby. …So that was a massive impact on us too. And [the baby] wasn't easy - she'd scream and cry and scream and cry because she didn’t know where she was and everyone was stressed around her. It was really hard.” (ID 09, child with end stage kidney failure) |
|  | “The other annoying thing is siblings can't come to appointments. So I've got a three year old, which is quite a problem, so I am constantly having to try and find people to mind him and put him in extra day care which is also money.” (ID 08, child with mid-aortic syndrome) |
| The impact of COVID-19 on support for families of children with chronic illness | |
| Summary | Quote |
| Family support became unavailable | “My mother-in-law said, ‘I’m happy to give you a break, come up and stay with him in hospital’. But because of COVID I just thought it was too risky. COVID’s been crap. It’s made things incredibly difficult. It’s made a really s**t situation even more difficult.” (ID 02, child with rhabdomyosarcoma) |
| Loss of support from charities | “This is also the first time we had to ever pay for accommodation in 6 years. My husband stays at Ronald McDonald House and I always stay with [my daughter]. So that was a massive impact on us too.” (ID 09, child with end stage kidney failure, husband had also lost his job in the pandemic). |
|  | “I think the biggest loss for my son was the fact that Starlight had closed. That was really the worst. I think the Starlight Captains pushed to be able to continue seeing the kids at least in the wards, which made a huge difference. I mean Starlight is so critical I think for all those kids.” (ID 02, child with rhabdomyosarcoma) |
| Support within the hospital | “So I think what’s been provided by the hospital in terms of support for the kids has been amazing, I can’t fault that, I think whatever was happening before, they really upped their game to keep that going so it all seems to be run really well.” (ID 11, child with acute lymphoblastic leukemia) |
| Social support | “I think people can understand a little bit more that fear that you do have as a chronic disease parent. Prior to COVID people have no clue what it is to be fearful for their child or their own health. …Now they've been faced with this fear that there is something out there that could kill our family. There have been a few comments from people like “Wow, how have you been able to do it, it's so scary and you live in such an uncertain way!”. And like in a funny way I think other people are a little bit more aware and thoughtful.” (ID 10, child with epilepsy) |
| COVID-19 interruptions to education | |
| Summary | Quote |
| Home-schooling period extended | “I could send him to school, but I’m just conscious of the fact that I don’t want him to catch COVID if that’s going to compromise any opportunity for surgery.” (ID 13, child with epilepsy (cortical dysplasia)) |
| Impact on siblings’ education | “I had to balance the need to protect [my child with a chronic illness] with his brother’s wellbeing. We kept [our healthy child] back from school and that was based on my anxiety, …but I got to a point where I thought there needs to be a balance between looking after his little brother and him. It’s not right for him to be home like this if everyone else is back.” (ID 02, child with rhabdomyosarcoma) |
| Limited teacher-parent communication | “I do wonder if it wasn’t a COVID environment …whether we’d have had more direct access to the teachers and [it would’ve been] easier to manage. I feel like even the sort of casual conversations like, ‘Has [my son] done anything unusual at school, have you seen any unusual behaviour?’ those sorts of conversations [would’ve been] different …if it wasn’t in a COVID environment.” (ID 13, child with epilepsy (cortical dysplasia)) |
| Burden of chronically ill children on limited staff | “I don’t feel like it’s appropriate for me to be putting a child with special needs in a school that’s got limited staff. I just think if he was going to have a seizure at school, which would normally happen every day before this recent lockdown, it’s just not fair on the teachers or the other kids.” (ID 13, child with epilepsy (cortical dysplasia)) |
| Pandemic initiatives supporting learning | “There's an organization through the US called Outschool [and] we started putting him in some classes. So all these little things that have sprung up have actually been really good.” (ID 04, child with acute lymphoblastic leukemia) |
| Pandemic impact impacted all children | “We are lucky that we had done distance education before …we sort of knew what was going on to an extent and were able to manage it. Whereas then I've talked to parents [of healthy children] and they are struggling because they’ve never had kids in the house so long!” (ID 10, child with epilepsy) |
| Hygiene at school | “Definitely the silver lining was having [my daughter] at school and knowing that it was a very clean environment and as safe as it probably could be. I also know that teachers were sending sick children home and parents were mostly keeping them at home. They're all using hand sanitizer. It’s almost the school made for [my daughter] at the moment.” (ID 09, child with end stage kidney failure) |
|  | “Especially at schools …I’m sure the children never used to wash their hands before, and now they have to several times a day. …It was really, really good in a way because every little viral illness can cause or trigger a relapse. [But] she hasn’t really been sick all year, so she hasn’t had a relapse for 6-7 months.” (ID 03, child with nephrotic syndrome) |
|  | “So a positive [was] last year we actually [were] stable medically. Just because of the lack of viral infections going around. Learning about hand washing at school, social distancing, everyone was wearing a mask, more acceptable classroom environment. So it just reduced the viral exposure, which meant we could commence with her treatment. So we just had a really good 12 months.” (ID 10, child with epilepsy) |
