# Supplementary material for: It’s made a really hard situation even more difficult: The impact of COVID-19 on families of children with chronic illness
Source: PLoS One. 2022 Sep 1;17(9):e0273622. doi: 10.1371/journal.pone.0273622 (PMC9436103; doi:10.1371/journal.pone.0273622)

PLOS ONE

It’s made a really hard situation even more difficult: The impact of COVID-19 on families of children with chronic illness

--Manuscript Draft--

Manuscript Number:	PONE-D-22-14320
Article Type:	Research Article
Full Title:	It’s made a really hard situation even more difficult: The impact of COVID-19 on families of children with chronic illness
Short Title:	The impact of COVID-19 on families of children with chronic illness
Corresponding Author:	Jordana McLoone, PhD<br>University of New South Wales - Kensington Campus: University of New South Wales<br>Kensington, New South Wales AUSTRALIA
Keywords:	COVID-19, pediatric, childhood chronic illness, psychosocial, qualitative
Abstract:	Objective: For over two years, the global COVID-19 pandemic has forced major transformations on ~~our~~ health, social, and educational systems, with concomitant impacts on mental health. This study aimed to understand the unique and additional challenges faced by children with chronic illness and their families during the COVID-19 era.<br>Method: Parents of children receiving treatment for a chronic illness within the neurology, cancer, renal and respiratory clinics of Sydney Children’s Hospital were invited to participate. We used qualitative methodology, including a semi-structured interview guide, verbatim transcription, and thematic analysis supported by QSR NVivo.<br>Results: Thirteen parents of children receiving tertiary-level care, for nine chronic illnesses, participated. Parents reported intense fears relating to their ill child’s additional vulnerabilities, which included their risk of developing severe COVID-19 disease and the potential impact of COVID-19-related disruptions to accessing clinical care, medications, allied health support and daily care protocols should their parent contract COVID-19. Parents perceived telehealth as a highly convenient and preferred method for ongoing management of less complex healthcare needs. Parents reported that the accrual of additional stressors and responsibilities during the pandemic, experienced in combination with restricted social interaction and reduced access to usual support networks was detrimental to their own mental health. Hospital-based visitation restrictions reduced emotional support, coping, and resilience for both parents and children and in some cases led to marital discord, sibling distress, and financial loss. Supportive factors included increased time spent together at home during the pandemic and improved hygiene practices at school, which dramatically reduced the incidence of non-COVID-19-related communicable illnesses in chronically ill children.<br>Discussion : For families caring for a chronically ill child, COVID-19 made a difficult situation harder. The pandemic has highlighted the need for targeted psychosocial intervention for vulnerable families, to mitigate current mental health burden and prevent chronic psychological distress.
Order of Authors:	Jordana McLoone, PhD
	Claire E Wakefield
	Glenn M Marshall
	Kristine Pierce
	Adam Jaffe
	Ann Bye
	Sean E Kennedy
	Donna Drew
	Raghu Lingam

Powered by Editorial Manager® and ProduXion Manager® from Aries Systems Corporation

Additional Information:	
**Question**	**Response**
**Financial Disclosure**<br><br>Enter a financial disclosure statement that describes the sources of funding for the work included in this submission. Review the submission guidelines for detailed requirements. View published research articles from *PLOS ONE* for specific examples.<br><br>This statement is required for submission and **will appear in the published article** if the submission is accepted. Please make sure it is accurate.<br><br>**Unfunded studies**<br>Enter: *The author(s) received no specific funding for this work.*<br><br>**Funded studies**<br>Enter a statement with the following details:<br>• Initials of the authors who received each award<br>• Grant numbers awarded to each author<br>• The full name of each funder<br>• URL of each funder website<br>• Did the sponsors or funders play any role in the study design, data collection and analysis, decision to publish, or preparation of the manuscript?<br>• **NO** - Include this sentence at the end of your statement: *The funders had no role in study design, data collection and analysis, decision to publish, or preparation of the manuscript.*<br>• **YES** - Specify the role(s) played.<br><br>* typeset	The author(s) received no specific funding for this work.
**Competing Interests**<br><br>Use the instructions below to enter a competing interest statement for this submission. On behalf of all authors, disclose any competing interests that could be perceived to bias this work—acknowledging all financial support and any other relevant financial or non-financial competing interests.	The authors have declared that no competing interests exist.

This statement is required for submission and **will appear in the published article** if the submission is accepted. Please make sure it is accurate and that any funding sources listed in your Funding Information later in the submission form are also declared in your Financial Disclosure statement.<br><br>View published research articles from *PLOS ONE* for specific examples.<br><br>**NO authors have competing interests**<br><br>Enter: *The authors have declared that no competing interests exist.*<br><br>**Authors with competing interests**<br><br>Enter competing interest details beginning with this statement:<br><br>*I have read the journal's policy and the authors of this manuscript have the following competing interests: [insert competing interests here]*<br><br>* typeset	
**Ethics Statement**<br><br>Enter an ethics statement for this submission. This statement is required if the study involved:<br><br>• Human participants<br>• Human specimens or tissue<br>• Vertebrate animals or cephalopods<br>• Vertebrate embryos or tissues<br>• Field research<br><br>Write "N/A" if the submission does not require an ethics statement.<br><br>General guidance is provided below. Consult the submission guidelines for detailed instructions. **Make sure that all information entered here is included in the**	The Sydney Children's Hospital Network ethics board granted ethical approval (2020/ETH02027). Participants provided written consent.

**Methods section of the manuscript.**<br><br>**Format for specific study types**<br><br>**Human Subject Research (involving human participants and/or tissue)**<br>• Give the name of the institutional review board or ethics committee that approved the study<br>• Include the approval number and/or a statement indicating approval of this research<br>• Indicate the form of consent obtained (written/oral) or the reason that consent was not obtained (e.g. the data were analyzed anonymously)<br><br>**Animal Research (involving vertebrate animals, embryos or tissues)**<br>• Provide the name of the Institutional Animal Care and Use Committee (IACUC) or other relevant ethics board that reviewed the study protocol, and indicate whether they approved this research or granted a formal waiver of ethical approval<br>• Include an approval number if one was obtained<br>• If the study involved *non-human primates*, add *additional details* about animal welfare and steps taken to ameliorate suffering<br>• If anesthesia, euthanasia, or any kind of animal sacrifice is part of the study, include briefly which substances and/or methods were applied<br><br>**Field Research**<br><br>Include the following details if this study involves the collection of plant, animal, or other materials from a natural setting:<br>• Field permit number<br>• Name of the institution or relevant body that granted permission	
**Data Availability**<br><br>Authors are required to make all data underlying the findings described fully available, without restriction, and from the time of publication. PLOS allows rare exceptions to address legal and ethical concerns. See the PLOS Data Policy and FAQ for detailed information.	No - some restrictions will apply

A Data Availability Statement describing where the data can be found is required at submission. Your answers to this question constitute the Data Availability Statement and **will be published in the article**, if accepted.<br><br>**Important:** Stating 'data available on request from the author' is not sufficient. If your data are only available upon request, select 'No' for the first question and explain your exceptional situation in the text box.<br><br>Do the authors confirm that all data underlying the findings described in their manuscript are fully available without restriction?	
**Describe where the data may be found in full sentences. If you are copying our sample text, replace any instances of XXX with the appropriate details.**<br><br>• If the data are **held or will be held in a public repository**, include URLs, accession numbers or DOIs. If this information will only be available after acceptance, indicate this by ticking the box below. For example: *All XXX files are available from the XXX database (accession number(s) XXX, XXX.*).<br>• If the data are all contained **within the manuscript and/or Supporting Information files**, enter the following: *All relevant data are within the manuscript and its Supporting Information files.*<br>• If neither of these applies but you are able to provide **details of access elsewhere**, with or without limitations, please do so. For example:<br><br>*Data cannot be shared publicly because of [XXX]. Data are available from the XXX Institutional Data Access / Ethics Committee (contact via XXX) for researchers who meet the criteria for access to confidential data.*<br><br>*The data underlying the results*	Data cannot be shared publicly in order to uphold the confidentiality of participants who have shared confidential medical information pertaining to children. Data are available from the corresponding author, provided written permission has been obtained from the named Ethics Committee.

presented in the study are available from (include the name of the third party and contact information or URL).<br>• This text is appropriate if the data are owned by a third party and authors do not have permission to share the data.<br><br>* typeset	
Additional data availability information:	

Manuscript

Click here to access/download;Manuscript;COVID and Chronic Illness 220517.docx

It's made a really hard situation even more difficult: The impact of COVID-19 on families of children with chronic illness

Short title: The impact of COVID-19 on families of children with chronic illness

Authors:

Jordana McLoone[1,2], Claire E.Wakefield[1,2], Glenn M. Marshall[3], Kristine Pierce[1], Adam Jaffe[1,4], Ann Bye[1], Sean E Kennedy[1,5], Donna Drew[3], and Raghu Lingam.[1]

Affiliations:

1. School of Clinical Medicine, UNSW Medicine and Health, UNSW Sydney, Sydney, New South Wales, Australia
2. Behavioural Sciences Unit, Kids Cancer Centre, Sydney Children's Hospital, Sydney, New South Wales, Australia
3. Kids Cancer Centre, Sydney Children's Hospital, Sydney, New South Wales, Australia
4. Respiratory Department, Sydney Children's Hospital, Randwick, New South Wales, Australia
5. Nephrology Department, Sydney Children's Hospital, Randwick, New South Wales, Australia

Corresponding author: Dr Jordana McLoone; J.McLoone@unsw.edu.au

Keywords:

COVID-19, pediatric, childhood chronic illness, psychosocial, qualitative

Abstract

Objective: For over two years, the global COVID-19 pandemic has forced major transformations on ~~our~~ health, social, and educational systems, with concomitant impacts on mental health. This study aimed to understand the unique and additional challenges faced by children with chronic illness and their families during the COVID-19 era.

Method: Parents of children receiving treatment for a chronic illness within the neurology, cancer, renal and respiratory clinics of Sydney Children's Hospital were invited to participate. We used qualitative methodology, including a semi-structured interview guide, verbatim transcription, and thematic analysis supported by QSR NVivo.

Results: Thirteen parents of children receiving tertiary-level care, for nine chronic illnesses, participated. Parents reported intense fears relating to their ill child's additional vulnerabilities, which included their risk of developing severe COVID-19 disease and the potential impact of COVID-19-related disruptions to accessing clinical care, medications, allied health support and daily care protocols should their parent contract COVID-19. Parents perceived telehealth as a highly convenient and preferred method for ongoing management of less complex healthcare needs. Parents reported that the accrual of additional stressors and responsibilities during the pandemic, experienced in combination with restricted social interaction and reduced access to usual support networks was detrimental to their own mental health. Hospital-based visitation restrictions reduced emotional support, coping, and resilience for both parents and children and in some cases led to marital discord, sibling distress, and financial loss. Supportive factors included increased time spent

together at home during the pandemic and improved hygiene practices at school, which dramatically reduced the incidence of non-COVID-19-related communicable illnesses in chronically ill children.

Discussion: For families caring for a chronically ill child, COVID-19 made a difficult situation harder. The pandemic has highlighted the need for targeted psychosocial intervention for vulnerable families, to mitigate current mental health burden and prevent chronic psychological distress.

Globally, COVID-19 has caused disruptions to the clinical management of chronic childhood illnesses, including reduced access to screening, diagnostic and therapeutic services, surgery, essential medications, and follow-up surveillance (1-4). Arising in parallel with the medical challenges of the pandemic are immense changes to the social landscape. The lifestyle and needs of the child, including education and peer interactions, have been curbed by social isolation and school closures (5-8). For children with a chronic illness, who already face a disproportionate level of psychosocial burden (9), it is expected that this confluence of factors may result in additional consequences compared to the general population (10).

Across childhood chronic illnesses, parents fill gaps in fragmented and uncoordinated healthcare systems, functioning as case managers, medical record keepers, and patient advocates. The pandemic required parents to rapidly navigate their child's new care pathways, as health systems became overwhelmed and healthcare delivery models pivoted to telehealth and virtual care (11, 12). Simultaneously, workplaces and schools also transitioned to virtual sites, with little time for planning, piloting, or training. Up-skilling to new work platforms, procedures, and home-learning responsibilities, while caring for a chronically ill child, is likely to have placed parents under unprecedented levels of pressure. Not surprisingly, emerging evidence indicates that parents of children with underlying health conditions have been experiencing greater stress than parents of healthy children during the pandemic (13, 14).

Given the vital role families play in the health of children with chronic illness, a deeper understanding of their pandemic experiences is needed to ensure that evidence-based research guides decision-makers as they rapidly develop and deploy

new services, and also re-imagine what a return to a new normal might look like for child healthcare services. This study therefore explored the impact of COVID-19 on families of children with chronic illness.

Method

We used an explorative, qualitative methodology to obtain nuanced insights into the experiences of children with chronic illness and their families during the COVID-19 pandemic. We first conducted a narrative literature review of the emerging literature on COVID-19 and its impact on children with disability or illness. This review was guided by the Economic and Social Research Council framework (15) and we presented this to a multidisciplinary panel, including five pediatric specialists, a behavioral scientist, and a parent consumer. The expert panel reviewed the literature and discussed the potential issues relevant to children with chronic illness in the context of the pandemic and developed a semi-structured interview guide through an iterative process. The final interview guide explored families' experiences of the COVID-19 pandemic and how their access to medical care, education, and social support had been impacted. The interview also explored the mental health impact of caring for a chronically ill child during a pandemic, as well as the mental health impacts for other family members, including the child with chronic illness and their sibling(s). At the conclusion of the interview we collected demographic items (e.g. age and sex of each family member).

First author JM (PhD), who has over a decade of qualitative research experience, interviewed families between November, 2020 and September, 2021. We audio-

recorded interviews, which were professionally transcribed verbatim. We organized coded passages into themes and analysis was guided by the Braun and Clarke approach (17). We used QSR NVivo 12 software to support data coding and analysis (18). We used the COREQ (19) checklist to promote accurate reporting of qualitative studies. The Sydney Children's Hospital Network ethics board granted ethical approval (2020/ETH02027).

Sample

We invited parents of children who a) were currently less than 16 years of age, b) had received treatment at Sydney Children's Hospital (SCH) within the past year, and c) were diagnosed with a chronic illness (a persistent illness requiring long term management), to participate in the study. SCH clinics including neurology, renal, respiratory and cancer, were asked to provide the contact details of families who met the study's eligibility criteria and were also purposively selected to provide a range of experiences, including recently diagnosed/long term patients, urban/rural, and severe/moderate disease. We sent families an invitation letter and consent form via mail and telephoned two weeks later to further explain the study and confirm whether or not a parent was interested in participating.

COVID-19 context

The COVID-19 context in which these interviews were conducted was as follows. Sydney Children's Hospital is located in the Australian state of New South Wales (NSW). NSW has a population of over 8 million residents, yet between 1 January 2020 and 1 June 2021, reported only 5,587 cases of COVID-19. There were 54 deaths during this period, with 13 of these deaths occurring outside of aged care services

[16]. For many months, there were zero to few cases of community transmission. Schools were “closed” for approximately six weeks, however the children of essential workers continued to be allowed to attend school during this period.

Results

Thirty families were invited to participate. Of these, 13 mothers participated (43% response rate), see Table 1. Interviews were, on average, 42 minutes in length (range: 17–61 minutes). Five major themes were identified; COVID-19 specific concerns and impacts on medical care, mental health, support, and education. These themes are explored in detail below and illustrated in Figure 1.

Fig 1: Complexity diagram representing the inter-relatedness of factors impacting families of children with chronic illness during the COVID-19 pandemic.

COVID-19 specific concerns

Parents reported that the pandemic had been a highly stressful period and described how, for their families, the pandemic stress was borne in addition to multiple other stressors. *“I mean things are already stressful in this house - a new baby and a chronically ill kid and you throw in COVID and job loss …things were intense a lot here.”* (ID 09, child with end stage kidney failure).

Parents of chronically ill children reported general feelings of uncertainty and fear, as well as fears unique to their child’s chronic health condition. *“I was terrified at the beginning of the pandemic. I just thought every time [my son] gets a cold he ends up*

in the hospital and I just thought I don't wanna think about what will happen if he gets this!" (ID 06, child with severe asthma). Parents' fears were typically intensified by extreme worries that the virus might have a greater impact on their chronically ill child, relative to children without chronic illness. *"I've watched her crash and go to ICU. You know what a virus can d*[illegible](ID 10, child with epilepsy). Parents of chronically ill children often reported taking precautions and self-restricting movement beyond what the Australian public health guidelines recommended and continued to do so during periods when there were no, or very few (<10), community cases in the state. *"I didn't go to the shops until Monday [December]. I hadn't been out like from February*[illegible](ID 09, child with end stage kidney failure).

Parents' fears centered on what might happen to their child if they became ill and could not support the daily medical needs of their child (see Table 2 for extended quotes). Parents worried that should a member of the family contract COVID-19, their child's chronic illness-related treatment would be postponed until the family completed their mandatory home-isolation period. *"That was my major anxiety last year, that one of us would get COVID and that would jeopardise his treatment. So that was my biggest concern*[illegible](ID 02, child with rhabdomyosarcoma). Families also reported fears that medical supplies might be disrupted during the pandemic. "*Because we rely on so many things to keep our little girl alive, I was definitely scared that things like that would be impacted."* (ID 09, child with end stage kidney failure) and in 2021, when the Delta strain caused case numbers to surge, parents were fearful that there would not be sufficient hospital beds available for their chronically ill child. *"I'm concerned if they [can] proceed with surgery that the beds*

will all be filled with anti-vaxxers and cases of COVID.” (ID 13, child with epilepsy (cortical dysplasia))

In terms of the emerging COVID-19 vaccines, some parents were concerned that there may be insufficient trialing of the vaccine in children with rare diseases, with potentially unknown risks. ‘I am also worried about vaccination because [my daughter] has a chronic illness. Like, is it going to be tested enough on people like that?” (ID 09, child with end stage kidney failure).

COVID-19 interruptions to medical care

Despite many challenges and disruptions, many families reported that their child’s acute care continued uninterrupted, and that telehealth had supported ongoing virtual clinics and communication between families of children with chronic illness and hospital clinicians. Many families felt that communication with their team had not been disrupted. *“The hospital team's been really supportive through it all. Always really impressed* - *we’ve been able to get onto them and everyone’s been really informative.”* (ID 10, child with epilepsy)

In terms of the ongoing management of chronic illnesses, some parents reported that clinics had been cancelle[illegible]*I think they cancelled all eye clinic reviews for us for nearly a year.”* (ID 10, child with epilepsy). In addition, certain medications had not been available at the hospital pharmacy. *“I get a phone call from pharmacy and she goes, “Look, you can’t take all the medication because you know, with COVID, we have a shortage.”* (ID 12, child with Dextrocardia situs inversus). There were also considerable delays reported regarding critical scans and surgeries. *“He actually*

needed surgery again but because of COVID …it was delayed for a long time. He was getting quite unwell and we just had to wait." (ID 08, child with mid-aortic syndrome)

Some families were burdened with the difficult decision of deciding whether or not to proceed with surgery, weighing the potential risks (exposure to COVID-19 within the hospital) and benefits (of surgery) for their child. Delays to surgery were often beyond the control of families and caused significant anxiety among parents, as they watched their child's health deteriorate. In addition, surgical delays often resulted in other developmental losses for young children, *"Huge worries about the future. [For] a 5 year old, a 6 months' delay [to surgery] is a huge portion of their life and it's such formative years in terms of their developmental opportunities."* (ID 13, child with epilepsy (cortical dysplasia)). For some children, accessing potentially life-altering surgery at centers of excellence remained uncertain due to the closure of state borders.

Clinicians used videoconferencing platforms to conduct virtual clinics, which were perceived as easy to use and highly convenient *"There are times [my daughter] might just not be feeling great, or we are worried because it[illegible]s winter …so we just do telehealth."* (ID 09, child with end stage kidney failure). The convenience of virtual clinics also included reducing travel burden, eliminating travel sickness, and fitting around other family commitments, for example, the schedules of a newborn baby. *"It's nice not having to drive from Canberra to Sydney [3-hour drive], as I had a little baby."* (ID 09, child with end stage kidney failure). Telehealth was seen as a tool that would also greatly benefit rural and regional families *"I think for regionally based*

people telehealth is great.” (ID 07, child with posterior urethral valves) and enhance interstate collaborations. Telehealth also supported the continuation of allied health services throughout the pandemic and families reported that this was convenient in many ways. *“The use of technology was forced upon everyone pretty quickly, but it certainly has its benefits.”* (ID 07, child with posterior urethral valves).

Families of children with chronic illness reported that their general practitioner’s (GP) use of telehealth was telephone-based (video-conferencing was not available) and that this this was the preferred method for low-risk, or ongoing management, for example, *“If it's something little that can be done over the phone, like a script or something”* (ID 03, child with nephrotic syndrome). Avoiding the contagion risks associated with the GP waiting room was also noted as a benefit of care provided via telehealth. *“I prefer not to take myself to a GP and risk catching something that I could give to [my chronically ill son] if I don't have to. So [I prefer] not taking my daughter there, or myself.”* (ID 04, child with acute lymphoblastic leukemia). Also, for families whose child was wheelchair bound, or who were less mobile due to their child’s chronic illness, telehealth with the GP was preferred. *“So it's made it so much easier, its brilliant actually its made life 100% better.”* (ID 01, child with cerebral palsy)

Given the convenience of telehealth, many families hoped that it would remain available as an option after the pandemic was over and cited advances in providing care remotely as a silver lining of the pandemic. The increased availability of and access to care that telehealth offers rural and remote families was also discussed. *“If we lived in a regional area [telehealth] would make a huge difference to accessing treatment.”* (ID 13, child with epilepsy (cortical dysplasia))

The impact of COVID-19 interruptions on mental health

Some families reported that they had been coping well during the pandemic. Protective factors included, higher resilience among families with a chronically ill child relative to other families; learning about self-care during earlier waves of the pandemic; and utilizing virtual support groups. "*I think there is a certain amount of resilience and adaptability that you have when you've got a child with a chronic illness.*" (ID 06, child with severe asthma). Many families reported that the ways in which the pandemic had forced them to slow down, limit extracurricular activities, and spend more time together as a family, had been beneficial. "*We were happy with just the four of us and our little family for a while.*" (ID 03, child with nephrotic syndrome)

Parents recognized how important their own mental health was during this difficult time, as they needed to stay strong for their sick child. *"I've got an added responsibility to manage my feelings… I don't want to impact his situation [and make it] more difficult because I'm not managing how I feel."* (ID 13, child with epilepsy (cortical dysplasia)). Parents noted that they were having to manage their own mental health needs as they journeyed through their child's illness path, "*The main thing that I've struggled with is sitting with the current uncertainty and trying to hold hope but also prepare myself for the possibility that it's not treatable. I feel more despair and more hopelessness. Looking after [my son] I guess I've got an added responsibility to manage my feelings. I don't want to impact his situation and make it more difficult because I'm not managing how I feel.*" (ID 13, child with epilepsy), as well as the needs of their chronically ill child *"It's a bit of a rollercoaster ride with her, still*

she's just sad." (ID 10, child with epilepsy), and siblings' mental health too, *"he's ended up with a bit of ongoing anxiety since lockdown; which we are still dealing with now."* (ID 06, child with severe asthma).

Mothers recognized how much strain they had experienced to support others in the family, especially under circumstances that saw them also juggling multiple practical roles. Many mothers reported limited support for themselves, including not being able to see, or physically touch their friends during the most difficult times. *"When you're upset or something terrible happens you would hug your friends. ...I stopped that altogether."* (ID 11, child with acute lymphoblastic leukemia). Some mothers reported that it had been difficult to lose their supportive work environment during this time and missed the reprieve that their career experiences gave them in a face-to-face work environment.

While some parents reported that they were aware that excellent support for their child was available via their child's hospital psychologist and social worker, "*I've spoken to [the clinical psychologist] a few times and she's amazing."* (ID 05, child with pre B acute lymhoblastic leukemia), others reported that there were barriers to receiving mental health support during the pandemic, especially in the community setting. The pandemic had increased the need for services, at a time when services were limited, causing a backlog in private mental health services and long waitlists.

Many families were separated by pandemic-restrictions and the negative impact of this was consistently reported. To limit COVID-19 transmission risk, the hospital implemented a 'one-parent, no siblings' rule for most months from March, 2020

onwards. While parents understood the hospital's policy was aimed at protecting its patients and staff, they also felt this policy placed families at risk of mental health impacts as it limited the emotional support they could provide each other at critical times. Parents emphasized the importance of communicating well with each-other, especially during traumatic periods. Parents reported difficulty communicating their own emotional needs, as well as distressing and complex medical information to the second parent who was not allowed to enter the hospital.

Although parents were appreciative of the circumstance, they consistently emphasized the negative impact of siblings being unable to see each-other during hospital admissions. "*I'd say that probably had the biggest impact on the family."* (ID 11, child with acute lymphoblastic leukemia). Mothers of young siblings (e.g. breast-feeding infants) also found the experience to be distressing. *"I'm breastfeeding and I'm always the one that stays with [my chronically ill daughter]; that actually stressed me out probably nearly more than anything."* (ID 09, child with end stage kidney failure). Though a sibling exemption for breast-feeding infants was granted, allowing infants to accompany the mother in the hospital, infants were not allowed in the intensive care unit, causing additional stress and costs. Managing outpatient appointments was similarly reported as challenging for families with young children, from both a practical and financial perspective.

The impact of COVID-19 on support for families of children with chronic illness

Families of children with a chronic illness reported that they typically sought support from extended family, however risks and restrictions during the pandemic meant that this was often not possible. Community organizations were also reported as a vital

support network for families. Unfortunately, many were limited in their capacity during the pandemic. The loss of charity funded accommodation, as well as social and psychological support, was keenly felt. Social support was reported as integral to well-being, with parents discussing how their local community, comprised of families of children without a chronic illness, suddenly became more understanding of what it was like to live with ongoing uncertainty and fear for their child's health. However, the valuable social support shared between families of children with a similar illness on the hospital wards was interrupted as many families tried to maintain social distancing and kitchens/communal areas were closed.

COVID-19 interruptions to education

Home-based-learning was seen as highly challenging during periods of school closures. *"I was literally ready to go 'See you all later, I am never coming back'. It was that bad."* (ID 12, child with Dextrocardia situs inversus). Families of children with a chronic illness also raised unique issues, such as worries that if they ceased home schooling and returned their child to school, the spread of COVID-19 through their child's school might delay their child's treatment or surgery. Some parents shared that they also kept siblings home even after schools were re-opened, potentially compromising their learning outcomes, due to fears that "catching something" would compromise the treatment protocols of their ill child.

Parents expressed that they felt the COVID-safe arrangements at the school, including not allowing parents on campus, meant that they were less able to communicate with teachers, resulting in missed feedback regarding their child's educational needs, and medical symptoms. Parents shared that they felt guilty and reluctant to send their

child with additional needs to school during “closures”, as schools had limited staff on campus.

Though parents often waited additional weeks before allowing their child to return to school after periods of closure, once most children had returned to school, the school stopped supporting home-based-learning leading to some parents feeling that their child was disadvantaged. However, other parents noted that the pandemic had actually helped their child who would not have been able to attend school due to illness, by increasing the availability of online learning platforms and resources. Some parents of chronically ill children also reported that they felt like they were better equipped to manage distance education than families of healthy children, due to past experiences.

Parents shared that they appreciated that schools and students were maintaining better cleaning and hygiene practices, and that children who were unwell or displaying any symptoms were discouraged from attending school. Parents noted that given these measures of prevention, their child had experienced fewer infections over the past several months with a notable positive impact on their health.

Discussion

Despite experiencing lower levels of COVID-19 than many other parts of the world, especially during 2020, disruptions to health, education and social support have had an acute psychosocial impact on Australian families of children with chronic illness. Adequately responding to and addressing the unique needs of these vulnerable families, as well as planning for the minimization of longer-term impacts is critical.

As hospitals rapidly reorganized in response to the developing pandemic, many clinics were temporarily closed and clinicians were challenged to find alternative ways to continue to provide treatment and care while maintaining social distancing practices. For most, embracing telehealth was the obvious solution and the rapid uptake of virtual care was supported by new videoconferencing platforms that patients and their families found easy to access and use (20). Positive attitudes towards pediatric telehealth (21, 22) are supported by our current study findings, with telehealth perceived to be convenient and one of the pandemic's "silver linings", which families hoped would remain after COVID-19 had abated. New models of care that integrate telehealth need to be developed, superseding the current model of repeatedly delivering stand-alone instances of use (i.e. the stop-gap use model) (23, 24). Comprehensive models that offer a number of services, increase in complexity as needed, and address needs from diagnosis to disease progression and long term care are required. Such programs are beginning to emerge, including Contactless (25), which specifically addresses the care needs of pediatric patients with rare and chronic conditions, who typically need regular follow-up even in the absence of acute events.

While telehealth has the potential to offer convenient care, improve access for rural families, and increase equity by decreasing the costs associated with accessing care for patients, more comprehensive evidence is needed to demonstrate that the telehealth model of care is at least equivalent to face to face care in terms of health outcomes. Also, telehealth may not be appropriate for families closer to diagnosis, whose child's management is still new, difficult, unfamiliar or fluctuating, and for families who are yet to establish a good working relationship with their clinical team.

Special consideration of these newer families’ needs should be taken into account when designing care pathways that incorporate or rely on telehealth.

Sydney Children’s Hospital Network has reported a 55% increase in pediatric mental health presentations during the COVID-19 period (26). The findings of this study confirm that there is a significant unmet need for psychosocial support among families of children with chronic illness. While this need most likely predated the pandemic, it is likely to have become stronger as stressors compound, services are stretched, and waitlists lengthen. This need for psychosocial support is reiterated in the emerging literature (1, 27-30) and new scales, such as the ‘COVID-19 Family Stressor Scale’ have been developed to measure COVID-related disruption and psychological stress (31). It is important to remember, as many countries re-establish unrestricted movement and confidence, that many families of children with chronic illness will continue to prioritize their child’s health over their family’s need to return to normal. Among a community already fatigued by multiple lockdowns and pandemic stress, these families may experience an extended period of stress, fear, restrictions and diminished social interaction. It is critical to acknowledge that families of children with chronic illness may have a disproportionately difficult experience relative to other groups. Ensuring that appropriate psychosocial support services are available to families of children with chronic illness is a critical part of our community's roadmap out of the COVID-19 pandemic.

Families of children with chronic illness reported hospital visitation restrictions as one of the most challenging aspects of the pandemic, both practically and emotionally. During the pandemic, pediatric wards have been challenged with unique

considerations in regards to visitation policies. While early evidence indicates visitor restriction is effective in preventing transmission of viruses within hospital settings (32), there are substantial complexities to be considered on pediatric wards. These include the dependence of young children on their parents for nutrition (e.g. breast feeding newborns), accomplishing daily-living tasks (e.g. dressing), decision-making (including medico-legal decisions), patient and parent education, and emotional comfort during traumatic illness and procedures.

Internationally, approximately 94% of North American hospitals with a pediatric ward, changed their visitor screening policy due to COVID-19 (33). Though limiting visitors to children's hospitals is consistent internationally, we call on researchers and clinicians to re-imagine infection control within a family-centered model of care (34) that will limit parent reported increases in stress, feelings of emotional isolation and decreased support, decreased medical communication, marital discord, sibling distress, financial stress, chronic sleep deprivation and career interruption. Additional psychosocial support to families on the wards should be provided until revised models of COVID-safe, family-centered care can be implemented.

Limitations

Our findings are based on the experiences of mothers only, with no fathers choosing to participate. Wade et. al. have shown that mothers have generally experienced greater COVID stress/disruption, distress, anxiety, and post-traumatic stress symptoms, compared to fathers. In the Wade et. al. study mothers' higher COVID stress/disruption independently predicted all mental health outcomes, suggesting a stress accumulation model (35). This is consistent with our finding that mothers

reported one of the most difficult aspects of the pandemic was the “juggling act” that they had to perform. It is also possible that hospitals’ one-parent rule acts as a protective factor among fathers who, in this way, are shielded from distressing scenes of their child being ill, difficult conversations with medical professionals, and are not burdened by multiple responsibilities. Further research is needed to explore the experiences of fathers specifically.

This study is also limited to English speaking participants, recruited from a single site, and the recruitment of a small sample. Though the sample size of this study is small, sample adequacy in qualitative investigation relates to the appropriateness of the sample composition and size (36). By purposively selecting families across a range of different diseases, ages, times since diagnosis, distances from the hospital and personal circumstances, the composition of this sample provides richly-textured and wide-ranging information relevant to the COVID-19 experience. Recent research has shown greater efficiency of purposive sampling (and accordingly lower sample size needs) compared to random sampling, as used in qualitative studies (37). Furthermore, data saturation was observed within this sample, with no new themes reported within the last two interviews.

Conclusions

The pandemic has, and will likely continue to go through waves, as new variants develop. It is critical that we understand the challenges and needs of families with chronically ill children and prepare a health and education system that will meet these needs in the future. Key lessons learned during the COVID-19 era can also inform

innovations that generalize beyond the pandemic, including the benefits of advanced telehealth for rural and unwell families, the importance of psychosocial and family-centered care.

Acknowledgements:

The authors acknowledge the support of the Maridulu Budyari Gumal, The Sydney Partnership for Health, Education, Research & Enterprise (SPHERE) Child Unlimited Clinical Academic Group; CEW is supported by the National Health and Medical Research Council of Australia (APP2008300).

References

1. Moreira DC, Millen GC, Sands S, Kearns PR, Hawkins DS. The Care of Children With Cancer During the COVID-19 Pandemic. American Society of Clinical Oncology Educational Book. 2021(41):e305-e14.
2. Tse Y, Darlington A-SE, Tyerman K, Wallace D, Pankhurst T, Chantziara S, et al. COVID-19: experiences of lockdown and support needs in children and young adults with kidney conditions. Pediatric Nephrology. 2021;36(9):2797-810.
3. Reilly C, Muggeridge A, Cross JH. The perceived impact of COVID-19 and associated restrictions on young people with epilepsy in the UK: Young people and caregiver survey. Seizure. 2021;85:111-4.
4. Boechat JL, Wandalsen GF, Kuschnir FC, Delgado L. COVID-19 and pediatric asthma: clinical and management challenges. International journal of environmental research and public health. 2021;18(3):1093.
5. Azevedo JP, Hasan A, Goldemberg D, Geven K, Iqbal SA. Simulating the potential impacts of COVID-19 school closures on schooling and learning outcomes: A set of global estimates. The World Bank Research Observer. 2021;36(1):1-40.
6. Engzell P, Frey A, Verhagen MD. Learning loss due to school closures during the COVID-19 pandemic. Proceedings of the National Academy of Sciences. 2021;118(17).
7. Fontenelle-Tereshchuk D. Mental health and the COVID-19 crisis: The hopes and concerns for children as schools re-open. Interchange. 2021;52(1):1-16.
8. Ye J. Pediatric mental and behavioral health in the period of quarantine and social distancing with COVID-19. JMIR pediatrics and parenting. 2020;3(2):e19867.

9. Mattson G, Kuo DZ, Yogman M, Baum R, Gambon TB, Lavin A, et al. Psychosocial factors in children and youth with special health care needs and their families. Pediatrics. 2019;143(1).

10. Serlachius A, Badawy SM, Thabrew H. Psychosocial challenges and opportunities for youth with chronic health conditions during the COVID-19 pandemic. JMIR Pediatrics and Parenting. 2020;3(2):e23057.

11. Edge R, Meyers J, Tiernan G, Li Z, Schiavuzzi A, Chan P, et al. Cancer care disruption and reorganisation during the COVID-19 pandemic in Australia: A patient, carer and healthcare worker perspective. PLOS ONE. 2021;16(9):e0257420.

12. Garfan S, Alamoodi AH, Zaidan BB, Al-Zobbi M, Hamid RA, Alwan JK, et al. Telehealth utilization during the Covid-19 pandemic: A systematic review. Computers in Biology and Medicine. 2021;138:104878.

13. Kostopoulou E, Sinopidis X, Gidaris D, Karantaglis N, Kassimos D, Gkentzi D, et al. Parents under siege: the psychological impact of COVID-19 outbreak on children's caregivers. Swiss Medical Weekly. 2021(31).

14. Wimberly CE, Towry L, Caudill C, Johnston EE, Walsh KM. Impacts of COVID-19 on caregivers of childhood cancer survivors. Pediatric Blood & Cancer. 2021;68(4):e28943.

15. Craig P, Dieppe P, Macintyre S, Michie S, Nazareth I, Petticrew M. Developing and evaluating complex interventions: the new Medical Research Council guidance. BMJ. 2008;337:a1655.

16. Australian Government DoH. Coronavirus (COVID-19) at a glance for 30 June 2021. https://wwwhealthgovau/resources/publications/coronavirus-covid-19-at-a-glance-30-june-2021. 2021.

17. Clarke V, Braun V. Thematic analysis. Encyclopedia of critical psychology: Springer; 2014. p. 1947-52.

18. QSR International Pty Ltd. (2020) NVivo (released in March 2020), https://www.qsrinternational.com/nvivo-qualitative-data-analysis-software/home.

19. Tong A, Sainsbury P, Craig J. Consolidated criteria for reporting qualitative research (COREQ): a 32-item checklist for interviews and focus groups. International journal for quality in health care. 2007;19(6):349-57.

20. Kenney LB, Vrooman LM, Lind ED, Brace-O'Neill J, Mulder JE, Nekhlyudov L, et al. Virtual visits as long-term follow-up care for childhood cancer survivors: Patient and provider satisfaction during the COVID-19 pandemic. Pediatric Blood & Cancer. 2021;68(6):e28927.

21. Rametta SC, Fridinger SE, Gonzalez AK, Xian J, Galer PD, Kaufman M, et al. Analyzing 2,589 child neurology telehealth encounters necessitated by the COVID-19 pandemic. Neurology. 2020;95(9):e1257-e66.

22. Dahl-Popolizio S, Carpenter H, Coronado M, Popolizio NJ, Swanson C. Telehealth for the Provision of Occupational Therapy: Reflections on Experiences During the COVID-19 Pandemic. Int J Telerehabil. 2020;12(2):77-92.

23. Camden C, Silva M. Pediatric Teleheath: Opportunities Created by the COVID-19 and Suggestions to Sustain Its Use to Support Families of Children with Disabilities. Physical & Occupational Therapy In Pediatrics. 2021;41(1):1-17.

24. Menon DU, Belcher HME. COVID-19 Pandemic Health Disparities and Pediatric Health Care—The Promise of Telehealth. JAMA Pediatrics. 2021;175(4):345-6.

25. Mercuri E, Zampino G, Morsella A, Pane M, Onesimo R, Angioletti C, et al. Contactless: a new personalised telehealth model in chronic pediatric diseases and disability during the COVID-19 era. Italian Journal of Pediatrics. 2021;47(1):29.

26. Hu N, Nassar N, Shrapnel J, Perkes I, Hodgins M, O'Leary F, et al. The impact of the COVID-19 pandemic on paediatric health service use within one year after the first pandemic outbreak in New South Wales Australia–a time series analysis. The Lancet Regional Health-Western Pacific. 2022;19:100311.

27. Durcan G, Barut K, Haslak F, Doktur H, Yildiz M, Adrovic A, et al. Psychosocial and clinical effects of the COVID-19 pandemic in patients with childhood rheumatic diseases and their parents. Rheumatology International. 2021;41(3):575-83.

28. Correale C, Tondo I, Falamesca C, Capitello TG, Vigevano F, Specchio N, et al. Depression and anxiety in hospitalized children with epilepsy during COVID-19 pandemic: Preliminary findings of a cross-sectional study. European Psychiatry. 2021;64(S1):S670-S1.

29. Farajzadeh A, Dehghanizadeh M, Maroufizadeh S, Amini M, Shamili A. Predictors of mental health among parents of children with cerebral palsy during the COVID-19 pandemic in Iran: A web-based cross-sectional study. Research in Developmental Disabilities. 2021;112:103890.

30. Havermans T, Houben J, Vermeulen F, Boon M, Proesmans M, Lorent N, et al. The impact of the COVID-19 pandemic on the emotional well-being and home treatment of Belgian patients with cystic fibrosis, including transplanted patients and paediatric patients. Journal of Cystic Fibrosis. 2020;19(6):880-7.

31. Prime H, Wade M, May SS, Jenkins JM, Browne DT. The COVID-19 Family Stressor Scale: validation and measurement invariance in female and male caregivers. Frontiers in Psychiatry. 2021;12.

32. Washam M, Woltmann J, Ankrum A, Connelly B. Association of visitation policy and health care-acquired respiratory viral infections in hospitalized children. American journal of infection control. 2018;46(3):353-5.

33. Kitano T, Piché-Renaud P-P, Groves HE, Streitenberger L, Freeman R, Science M. Visitor Restriction Policy on Pediatric Wards During Novel Coronavirus (COVID-19) Outbreak: A Survey Study Across North America. Journal of the Pediatric Infectious Diseases Society. 2020;9(6):766-8.

34. Goga A, Feucht U, Pillay S, Reubenson G, Jeena P, Madhi S, et al. Parental access to hospitalised children during infectious disease pandemics such as COVID-19. South African Medical Journal. 2021;111(2):100-5.

35. Wade M, Prime H, Johnson D, May SS, Jenkins JM, Browne DT. The disparate impact of COVID-19 on the mental health of female and male caregivers. Social Science & Medicine. 2021;275:113801.

36. Vasileiou K, Barnett J, Thorpe S, Young T. Characterising and justifying sample size sufficiency in interview-based studies: systematic analysis of qualitative health research over a 15-year period. BMC medical research methodology. 2018;18(1):1-18.

37. van Rijnsoever FJ. (I Can't Get No) Saturation: A simulation and guidelines for sample sizes in qualitative research. PloS one. 2017;12(7):e0181689.

Tables and figures

Table 1: Participant demographic characteristics

Participant characteristic	N=13
Parent sex, n(%)	13 female (100%)
Parent age, mean (range)	42.3 years (range: 36-52 years)
Sex of child with chronic illness, n(%)	61.5% male
Age of child with chronic illness, mean (range)	8.5 years (range: 4-16 years)
Number of siblings of child with chronic illness, mean (range)	1.8 siblings (range: 1-7)
Sibling age, mean (range)	12.5 years (range: 0-24 years)
Chronic illness diagnosis*	Cancer<br>Pre B Acute Lymphoblastic Leukemia<br>Acute Lymphoblastic Leukemia<br>Acute Lymphoblastic Leukemia<br>Rhabdomyosarcoma<br>Renal<br>Nephrotic syndrome<br>Posterior urethral valves<br>End stage kidney failure<br>Mid-aortic syndrome<br>Neurology<br>Epilepsy<br>Epilepsy (neuronal migration disorder)

	Epilepsy (cortical dysplasia)
	Other
	Cerebral Palsy
	Severe asthma
	Bronchiectesis
	Dextrocardia situs inversus

* Some children were diagnosed with multiple chronic illnesses and as such, more than 13 illnesses are reported.

Tabl[illegible] The perceived impact of COVID-19 - illustrative quotations of longer length.

COVID-19 specific concerns	
Summary	Quote
The severe impact of viruses on a chronically ill children	“Even as news came in that kids don’t seem to get sick with it so much, there was enough data online that said most kids don’t, but some kids do... So that was a really nerve wrecking experience.” (ID 06, child with severe asthma)
Prolonged isolation	We're more aware of where we’re going and how we’re acting in the community. We are very conscious and we’ve been masking for a long time. We've still got the hand sanitiser everywhere and we are still being cautious.” (ID 10, child with epilepsy)
The impact of parents’ developing COVID-19 on the child’s care needs	“I am very, very worried about if [my husband] and I got it - what would happen to [my daughter]. We do all of [her] care - her 11 hours of dialysis every night, we have to count every milliliter she's drinking, we have to double boil her puree, we have to weigh all her food, [illegible]xtreme okay and I am extremely scared about what would happen, if [my husband] and I went down - what would happen to [my daughter]?” (ID 09, child with end stage kidney failure)
	You know as a parent …they need you. Like it doesn’t work if we don’t have parents to keep this going forward. [illegible]hat risk, even if she’d be fine if she got it, if one of us got it, it would be disastrous.” (ID 10, child with epilepsy)

Chronically ill children advocated for the vaccine	"I think she was the most excited I've ever seen a child about anything when we got our vaccination appointment. She was really excited because in her mind that was a big thing to sort of mark …that it will be over." (ID 10, child with epilepsy)
COVID-19 interruptions to medical care	
Summary	Quote
Cancellation of clinics	"His last two operations they thought he had an anaphylactic reaction. So [he had an] allergy clinic [in] March, so they cancelled it then they made a new one, they cancelled it again… Then he had to have surgery in the beginning of August …and they wanted to do all the [allergy] testing before that, but they decided not to because of COVID. But then he had the reaction again! So then they decided, "Oh actually, you do need to come to [the allergy] clinic!" (ID 08, child with mid-aortic syndrome)
Delays for critical scans and surgeries	"They said that there was a big delay on being able to provide that [SPECT scan] because the radioactive substance that's injected in the kids, there was a shortage of that in Australia because of COVID." (ID 13, child with epilepsy (cortical dysplasia))
	"[the surgeon said] 'What do you want to do? We could probably squeeze her in before elective surgeries and all those things are closed and cancelled [end of March]. But obviously we've got the worry of COVID. What do you want to do?' And I'm like I don't know? It's a very hard decision

	to make. I really agonised over it and in the end … it got postponed. …The option [didn't become available again until] July. If this [surgery] had worked, [my daughter] would be getting a transplant." (ID 09, child with end stage kidney failure)
Interstate border closures restricted surgeries at centers of excellence	"When we spoke to them about the possibility of having surgery interstate, the body language of the doctor was very defensive. Whereas I think if it wasn't for COVID we would be able to access that service much more easily." (ID 13, child with epilepsy (cortical dysplasia))
Telehealth was more convenient	"We've had neurology, respiratory, bronchiectasis, pediatrics, all on telehealth. And it's been great. Yes. Really good. So much easier for me and for him because it really gets very draining going into the hospital for all of the appointments all of the time." (ID01, child with cerebral palsy)
Telehealth supported interstate collaborations for complex cases	"So all the appointments with the Melbourne doctors have been by telehealth, which has been great. So there's been a really nice collaboration between the Sydney doctors and the Melbourne doctors in terms of sharing of resources and that's definitely been amazing to have access to that service via telehealth." (ID 13, child with epilepsy (cortical dysplasia))
	"We were able to pivot her physio and do [it] by telehealth, which worked really well. Also, if we go to speech therapy we drive half an hour each direction, which means she [misses an] hour of school. If we can do the telehealth at 8

	o'clock in the morning just before school, it fits in a lot better with the day.” (ID 10, child with epilepsy)
Support for GP telehealth use	“For me I think telehealth was another silver lining… I think that’s been really good and that should stay because for people in my situation, not having to be physically present all the time is a blessing, whether or not there's a pandemic.” (ID 04, child with acute lymphoblastic leukemia)
The impact of COVID-19 interruptions on mental health	
Summary	Quote
Learning from experience	“This time around I’ve been able to manage [my mental health] a bit better because I’ve been able to put different things in place. I’ve actually taken more self care.” (ID 13, child with epilepsy (cortical dysplasia))
Peer support	“Some things sprung up like Redkite started doing online coffee groups and for a while I was joining that every week. That was actually quite good for me, quite therapeutic And also the Leukaemia Foundation did some similar kind of thing. So those were actually quite good for me in that lockdown, uncertain period[illegible]ID 04, child with acute lymphoblastic leukemia)
Increased family time	“Probably one of the positive things was that things slowed down quite a bit, so we weren’t rushing to afterschool activities and there was time for us to go on family walks, family bike rides and all that sort of stuff. So that was

	definitely a positive of [the pandemic].” (ID 07, child with posterior urethral valves)
	“We are just a tight knit family, so we got on really well. …We just adapted really well. At one stage [her brother] came up to me and was like, “This is really nice, she hasn’t been to the hospital for ages and we have all this time together”. So for them, they built this beautiful little relationship they'd not necessarily had the time together to develop because he's at school and she's just been out of the house. So… its quite nice because we finally got to spend time as a family.” (ID 10, child with epilepsy)
The multiple responsibilities of mothers	“I guess the hardest thing has just been the juggling act. Just the challenge has been trying to do my job, keep the business running, run a family, and now without any kind of babysitters or cleaners or any extra help and without school and without childcare, so that’s been the trickiest part of COVID I think for me personally.” (ID 13, child with epilepsy (cortical dysplasia))
	“I think because like a lot of families the burden of childcare has largely or exclusively fallen on the mother I’d say. So I’d say that’s mostly me that is suffering as a result of that, either the extra workload or just the awareness that the general sort of structure that I prefer to have within our family and within our life there’s had to be lots of compromises in terms of just logistically being able to juggle the competing

	responsibilities.” (ID 13, child with epilepsy (cortical dysplasia))
Distance from friends at challenging times	“[My daughter’s] initial [cancer] diagnosis was when we were really in the middle of the pandemic. When you're upset or something terrible happens you would hug your friends. …I stopped that altogether. I had friends dropping things off to me at the hospital and they’re like I wanna give you a hug and I’m like we’re not. I don’t want you. I think personally that affected me.” (ID 11, child with acute lymphoblastic leukemia)
Loss of professional identity	“When you get out of the house and you go to work, you're someone different, you're the person at work. But when you're kind of just stuck in this house with a pandemic, [your child’s illness] just becomes your identity and you're a cancer parent. The mental health impact of that was a lot greater than I recognized at the time.” (ID 04, child with acute lymphoblastic leukemia)
Psychosocial support was lacking	“I so badly need to see a counsellor. I really needed to spend some time talking to somebody particularly at the beginning. Like at the beginning they didn’t even want the social workers to meet us in person and it’s just ridiculous. Support staff needs to be accessible, if social workers and psych and things are not around it can have a terrible impact at certain times.” (ID 05, child with pre B acute lymhoblastic leukemia)

Long waitlists for psychosocial support	“We're finding now waitlists are a lot longer than normal for a lot of the [psychologists], just because they've got that backlog. It makes it a little trickier to access those services as a family. …it just takes a lot of running around to try and find places with availability.” (ID 10, child with epilepsy)
Mental health impact of the ‘one parent, no siblings’ hospital restrictions	“Hospitals are doing what they need to do, but there's a real risk attached to it. There's a real risk and I know mental health is the problem of 2020 for everybody, but there really is such a risk attached.” (ID 05, child with pre B acute lymhoblastic leukemia)
Separation of parents	“When my daughter was diagnosed [with cancer] they wouldn’t let my husband come into the hospital. He had to go downstairs and stand next to a Coke machine and listen in on a call with six people discussing my daughter’s diagnosis. And there was one afternoon she almost died - like the full-on warning. The professor was in the room like, "I’m concerned, you must prepare" and like my husband is sitting down in the car park. And he couldn’t support me emotionally because he just didn’t understand where I was at with it all because he just wasn’t in the room.” (ID 05, child with pre B acute lymhoblastic leukemia)
	“One of the biggest things [for] people with long term illness is you've got to stay on-board with your partner. Like the strain on your home-life is a very close second to the illness itself. [I was] sitting there crying outside the hospital saying

	to my husband this is a really serious moment and things are not matching and they are telling us… the conversations are starting…, and he was just oblivious to it. My husband's a reasonable man, I mean we have a good marriage, good communication, and we've mainly done well through this, but I just thought holy s**t, like at that moment if we weren't educated people, if English wasn't our first language, if there was any disadvantage, I don't know what could've happened that day." (ID 05, child with pre B acute lymhoblastic leukemia)
	"A lot of things get lost in translation …and it actually builds frustration between us. There's a lot of complex information that goes back and forward on a day to day basis that doesn't always get communicated because he is not there." (ID 11, child with acute lymphoblastic leukemia)
Separation of siblings	"They love each other. So she would cry for him and he was very, very confused because of his age [4 years], and he needed to see her. When she did see him, either coming home briefly or through the doors at the hospital, her mental state was immediately better for two or three days. The boost was really noticeable and it really helped our son to settle, because …he was just very confused and very upset. He was quite problematic." (ID 05, child with pre B acute lymhoblastic leukemia)

	"The kids are only 14 months apart so they are exceptionally close. [My daughter] is desperate for his companionship. She's just grown up with him that's all she's known. So for him not being able to come into the hospital, she has been incredibly distraught. I'd say that probably had the biggest impact on the family. Because I spend so much time in hospital, this past six months have been really traumatic for [my daughter], but the gravitating impact of that has been on his emotional well being. We'll have this connect-disconnect-connect-disconnect for him. So we've [had] unexpected behaviors from him we've put down to us not being together as a family and him being shuffled around quite a bit." (ID 11, child with acute lymphoblastic leukemia)
	They're the best of friends. And that was the other thing I thought - how can we say this little girl can't see her baby sister while she's going through such a hell time in the hospital? And again, I understand, at the same time I know COVID is scary and they have to make sure everyone's safe, but you also got to think about people and family. And given [my daughter's] trauma like, [her sibling] just calmed her right down. (ID 09, child with end stage kidney failure)
Challenges caring for young siblings hospital	"My husband and I were allowed in ICU …the baby [was] not allowed in ICU. We had to basically pay for my parents to stay in accommodation with us so they could help look after the baby. …So that was a massive impact on us too.

	And [the baby] wasn't easy - she'd scream and cry and scream and cry because she didn't know where she was and everyone was stressed around her. It was really hard." (ID 09, child with end stage kidney failure)
	"The other annoying thing is siblings can't come to appointments. So I've got a three year old, which is quite a problem, so I am constantly having to try and find people to mind him and put him in extra day care which is also money." (ID 08, child with mid-aortic syndrome)
The impact of COVID-19 on support for families of children with chronic illness	
Summary	Quote
Family support became unavailable	"My mother-in-law said, 'I'm happy to give you a break, come up and stay with him in hospital'. But because of COVID I just thought it was too risky. COVID's been crap. It's made things incredibly difficult. It's made a really s**t situation even more difficult." (ID 02, child with rhabdomyosarcoma)
Loss of support from charities	"This is also the first time we had to ever pay for accommodation in 6 years. My husband stays at Ronald McDonald House and I always stay with [my daughter]. So that was a massive impact on us too." (ID 09, child with end stage kidney failure, husband had also lost his job in the pandemic).
	"I think the biggest loss for my son was the fact that Starlight had closed. That was really the worst. I think the Starlight

	Captains pushed to be able to continue seeing the kids at least in the wards, which made a huge difference. I mean Starlight is so critical I think for all those kids." (ID 02, child with rhabdomyosarcoma)
Support within the hospital	"So I think what's been provided by the hospital in terms of support for the kids has been amazing, I can't fault that, I think whatever was happening before, they really upped their game to keep that going so it all seems to be run really well." (ID 11, child with acute lymphoblastic leukemia)
Social support	"I think people can understand a little bit more that fear that you do have as a chronic disease parent. Prior to COVID people have no clue what it is to be fearful for their child or their own health. …Now they've been faced with this fear that there is something out there that could kill our family. There have been a few comments from people like "Wow, how have you been able to do it, it's so scary and you live in such an uncertain way!". And like in a funny way I think other people are a little bit more aware and thoughtful." (ID 10, child with epilepsy)
COVID-19 interruptions to education	
Summary	Quote
Home-schooling period extended	"I could send him to school, but I'm just conscious of the fact that I don't want him to catch COVID if that's going to compromise any opportunity for surgery." (ID 13, child with epilepsy (cortical dysplasia))

Impact on siblings' education	"I had to balance the need to protect [my child with a chronic illness] with his brother's wellbeing. We kept [our healthy child] back from school and that was based on my anxiety, …but I got to a point where I thought there needs to be a balance between looking after his little brother and him. It's not right for him to be home like this if everyone else is back." (ID 02, child with rhabdomyosarcoma)
Limited teacher-parent communication	"I do wonder if it wasn't a COVID environment …whether we'd have had more direct access to the teachers and [it would've been] easier to manage. I feel like even the sort of casual conversations like, 'Has [my son] done anything unusual at school, have you seen any unusual behaviour?' those sorts of conversations [would've been] different …if it wasn't in a COVID environment." (ID 13, child with epilepsy (cortical dysplasia))
Burden of chronically ill children on limited staff	"I don't feel like it's appropriate for me to be putting a child with special needs in a school that's got limited staff. I just think if he was going to have a seizure at school, which would normally happen every day before this recent lockdown, it's just not fair on the teachers or the other kids." (ID 13, child with epilepsy (cortical dysplasia))
Pandemic initiatives supporting learning	"There's an organization through the US called Outschool [and] we started putting him in some classes. So all these little things that have sprung up have actually been really good." (ID 04, child with acute lymphoblastic leukemia)

Pandemic impact impacted all children	"We are lucky that we had done distance education before …we sort of knew what was going on to an extent and were able to manage it. Whereas then I've talked to parents [of healthy children] and they are struggling because they've never had kids in the house so long!" (ID 10, child with epilepsy)
Hygiene at school	"Definitely the silver lining was having [my daughter] at school and knowing that it was a very clean environment and as safe as it probably could be. I also know that teachers were sending sick children home and parents were mostly keeping them at home. They're all using hand sanitizer. Its almost the school made for [my daughter] at the moment." (ID 09, child with end stage kidney failure)
	"Especially at schools …I'm sure the children never used to wash their hands before, and now they have to several times a day. …It was really, really good in a way because every little viral illness can cause or trigger a relapse. [But] she hasn't really been sick all year, so she hasn't had a relapse for 6-7 months." (ID 03, child with nephrotic syndrome)
	"So a positive [was] last year we actually [were] stable medically. Just because of the lack of viral infections going around. Learning about hand washing at school, social distancing, everyone was wearing a mask, more acceptable classroom environment. So it just reduced the viral exposure, which meant we could commence with her treatment. So we

	just had a really good 12 months.” (ID 10, child with epilepsy)

Figure

Click here to access/download;Figure;Fig 1.tif

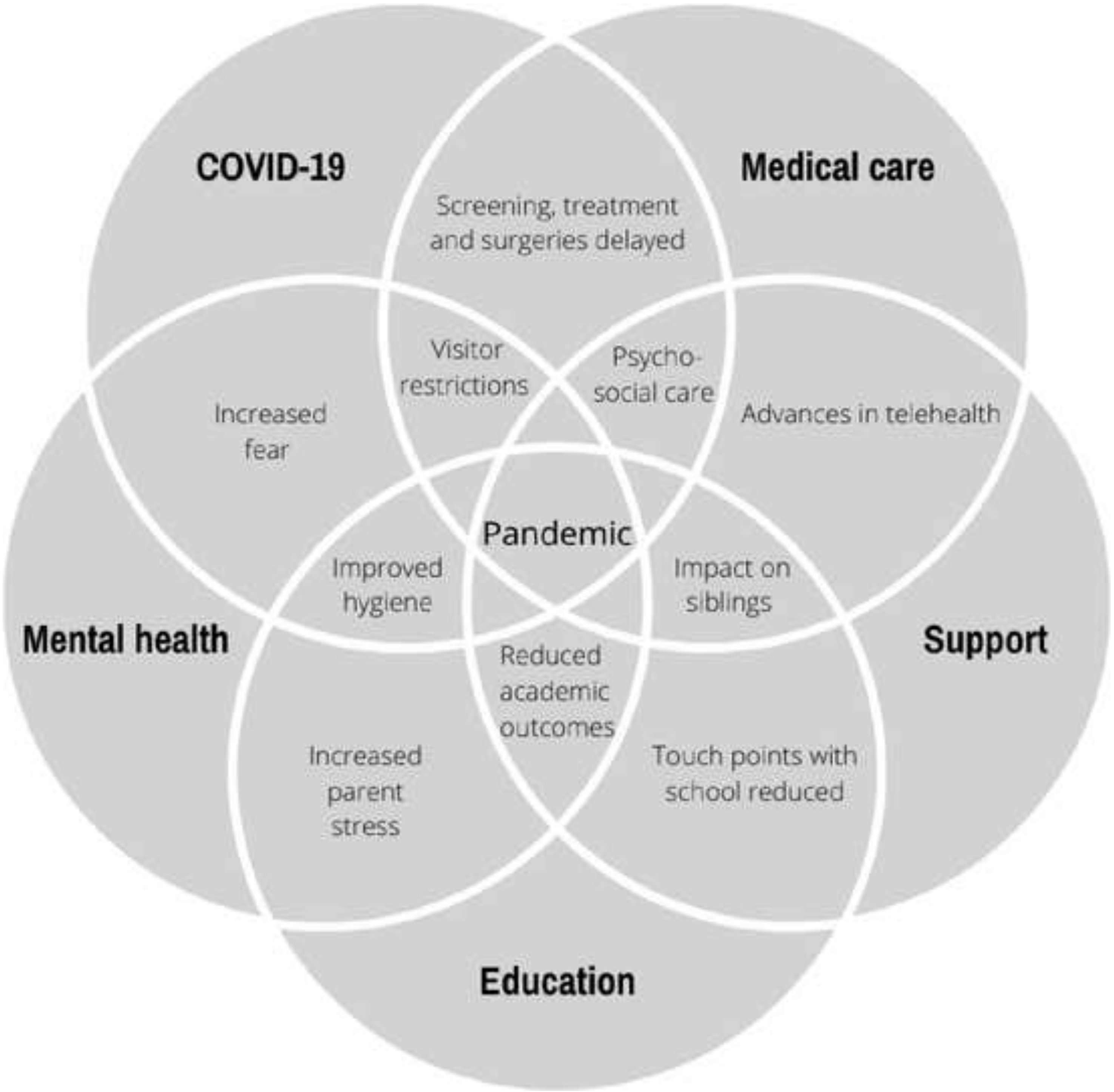

Supplement: Attachment — Submitted filename: PONE-D-22-14320_reviewer-bc.pdf [file pone.0273622.s002.pdf]
